# Artificial intelligence in sport: Exploring the potential of using ChatGPT in resistance training prescription

**DOI:** 10.5114/biolsport.2024.132987

**Published:** 2023-11-20

**Authors:** Jad Adrian Washif, Jeffrey Pagaduan, Carl James, Ismail Dergaa, Christopher Martyn Beaven

**Affiliations:** 1 Sports Performance Division, National Sports Institute of Malaysia, Kuala Lumpur, Malaysia; 2 Institute of Active Lifestyle, Palacký University Olomouc, Czech Republic; 3 Department of Sport, Physical Education and Health, Hong Kong Baptist University. Kowloon Tong, Hong Kong SAR; 4 Primary Health Care Corporation (PHCC), Doha, Qatar; 5 High Institute of Sport and Physical Education, University of Sfax, Sfax, Tunisia; 6 Te Huataki Waiora School of Health, University of Waikato, Tauranga, New Zealand

**Keywords:** Chatbot, Exercise prescription, Individualised training, Periodisation, Programming, Strength training

## Abstract

OpenAI’s Chat Generative Pre-trained Transformer (ChatGPT) technology enables conversational interactions with applications across various fields, including sport. Here, ChatGPT’s proficiency in designing a 12-week resistance training programme, following specific prompts, was investigated. GPT3.5 and GPT4.0 versions were requested to design 12-week resistance training programmes for male and female hypothetical subjects (20-years-old, no injury, and ‘intermediate’ resistance training experience). Subsequently, GPT4.0 was requested to design an ‘advanced’ training programme for the same profiles. The proposed training programmes were compared with established guidelines and literature (e.g., National Strength and Conditioning Association textbook), and discussed. ChatGPT suggested 12-week training programmes comprising three, 4-week phases, each with different objectives (e.g., hypertrophy/strength). GPT3.5 proposed a weekly frequency of ~3 sessions, load intensity of 70-85% of one repetition-maximum, repetition range of 4-8 (2-4 sets), and tempo of 2/0/2 (eccentric/pause/concentric/‘pause’). GPT4.0 proposed intermediate- and advanced programme, with a frequency of 5 or 4 sessions, 60-90% or 70-95% intensity, 3-5 sets or 3-6 sets, 5-12 or 3-12 repetitions, respectively. GPT3.5 proposed rest intervals of 90-120 s, and exercise tempo of 2/0/2. GPT4.0 proposed 60-180 (intermediate) or 60-300 s (advanced), with exercise tempo of 2/1/2 for intermediates, and 3/0/1/0, 2/0/1/0, and 1/0/1/0 for advanced programmes. All derived programmes were objectively similar regardless of sex. ChatGPT generated training programmes which likely require additional fine-tuning before application. GPT4.0 synthesised more information than GPT3.5 in response to the prompt, and demonstrated recognition awareness of training experience (intermediate vs advanced). ChatGPT may serve as a complementary tool for writing ‘draft’ programme, but likely requires human expertise to maximise training programme effectiveness.

## INTRODUCTION

Generative Pre-training Transformer (GPT) from OpenAI is a language model that attracted 1 million users within 5 days of launching the free model 3.5 (GPT3.5) on November 30, 2022 (https://openai.com/). Its paid successor, GPT4.0, launched on March 14, 2023 (https://openai.com/), enhanced its suitability for tasks requiring advanced reasoning. Based on Natural Language Processing, GPT generates human-like conversations through applications (chatbots or ChatGPT), providing contextually accurate responses to users’ inputs [1]. As such, ChatGPT has the potential to offer support in various fields, including academia and sport [2].

In academia, chatbots may serve as a “research assistant” to generate ideas, receive feedback, and summarise literature [2, 3, 4]. In sports, ChatGPT can produce training prescriptions, including plans, suggestions, and performance feedback based on specified information [2]. This is particularly useful in situations like Coronavirus disease 2019, where training support may be scarce [5]. In preparing training programmes, coaches utilise books, scholarly articles, and online resources [6]. Yet, the wealth of information, coupled with biases inherent in diverse data sources (e.g., erroneous conclusions or perceptions), remains challenging and time consuming to navigate. Globally, GPT technologies appear to offer an increasingly popular approach to streamlining such information gathering and synthesis. However, reported examples of its use in sport and exercise are scarce [2, 3].

Pre-trained on a large corpus of online data, the accuracy of ChatGPT in exercise prescription is not fully understood [7]. Such limitations must be recognised before the replacement of human intelligence can be countenanced in this area, through widespread adoption across the general population. Thus, this ‘short communication’ aimed to (i) explore and inform readers about ChatGPT technology in sports and exercise, (ii) highlight the potential use of ChatGPT for exercise prescription in resistance training, and (iii) compare the resistance training programmes designed by GPT3.5 and GPT4.0 in a hypothetical male and female participant.

## MATERIALS AND METHODS

In this study, we assessed ChatGPT’s ability to prescribe training for hypothetical individuals characterised as either intermediate (moderately resistance-trained) or advanced (well resistance-trained) via a series of prompts. The training programmes proposed by ChatGPT were carefully compared with authoritative literature, including the textbook of the National Strength and Conditioning Association (NSCA) and various review papers. The authors had expertise in sports science and exercise prescription; possessed strength and conditioning qualifications; and had 10–20 years of experience in designing and assessing resistance training programmes for athletes of varying experience levels, ranging from adolescent to Olympic. These backgrounds may add a valuable perspective to the appraisal (Table 1).

**TABLE 1:** Summary of the appraisal between and ChatGPT generated training programmes and scientific literature

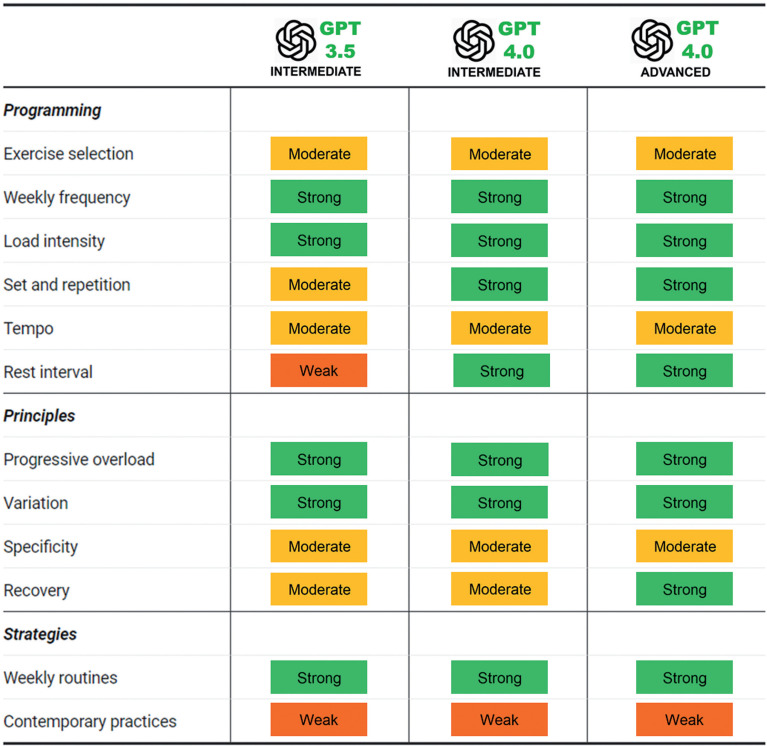

Note: Ratings are classified as ‘strong’, ‘moderate’, or ‘weak’, which are assigned by considering the appropriateness and completeness of how particular variables are integrated within the overall programme, including the degree to which the variables align with established scientific standards and practices.

### Exercise prescription using ChatGPT

**FIG. 1:**
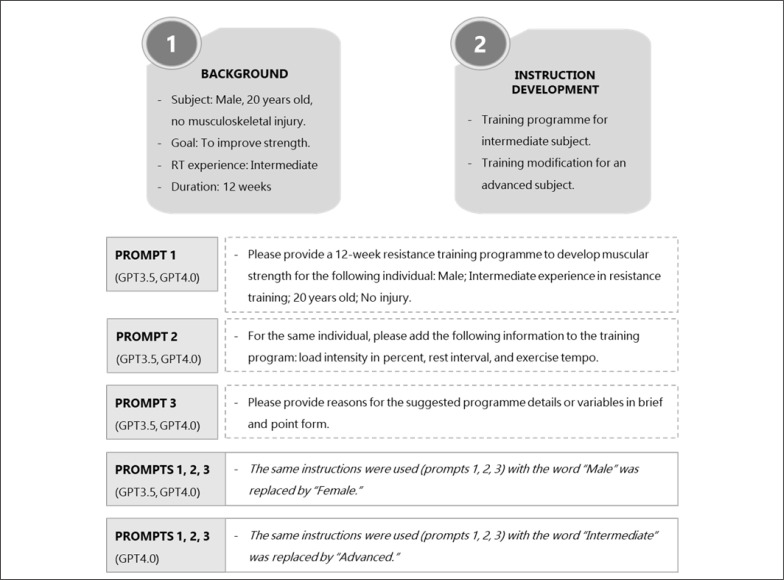
Prompts or instructions used to generate training programmes for intermediate (GPT3.5 and GPT4.0_INT_) and advanced (GPT4.0_ADV_) resistance training in male and female subjects.

Separately, we requested GPT3.5 and GPT4.0 (July 20, 2023 versions; OpenAI, L.L.C., San Francisco, CA, USA) to provide a 12-week resistance training programme to develop muscular strength, i.e., intermediate-level training programmes (abbreviated as GPT3.5 and GPT4.0_INT_ for each version, respectively). Technologies auto-generated texts based on three prompts (Figure 1). The texts (training programmes) generated by ChatGPT were transformed or condensed (manually) into table format to facilitate review and appraisal (supplementary file 1 [S1], and supplementary file 2 [S2]). Our preliminary prompts yielded comparable recommendations for both the male and female; therefore, we only report male responses in this brief report. Subsequently, we asked GPT4.0 to create an advanced training programme (GPT4.0_ADV_) for the same participants, using the same prompts except for the training level (Figure 1).

## RESULTS

A summary of ChatGPT’s responses (Table 1), the requested 12-week resistance training programmes (S1) and reasoning (S2), are provided. Briefly, both GPT3.5 and GPT4.0 proposed three periodised phases of training (foundation *and* high volume; strength building *and* moderate volume/high intensity; and intensification *and* low volume/very high intensity) lasting 4 weeks. The advanced programme also utilised 4-week phases, but proposed block periodisation (accumulation, intensification, and realisation). Preliminary analysis specific to “female prompts” revealed objectively similar training recommendations for both male and female participants.

For *(i) exercise selection and structure*, ChatGPT recommended exercises in two (GPT3.5 and GPT4.0_ADV_) or three (GPT4.0_INT_) exercise groups, using a split-routine approach. For *(ii) training frequency,* ~3 sessions/week (GPT3.5), 4 sessions/week (GPT4.0_ADV_), and 5 sessions/week (GPT4.0_INT_), were proposed. For (*iii*) *load intensity*, GPT3.5 (70–75% to 75–80% to 80–85%), GPT4.0_INT_ (60–70% to 70–80% to 80–90%), and GPT4.0_ADV_ (70–80% to 80–90% to 90–95%) provided subtly different progressions. For *(iv) sets and repetitions*, GPT3.5 proposed 2–4 sets and 4–8 repetitions for most exercises, while GPT4.0_INT_ and GPT4.0_ADV_ proposed 3–5 sets and 5–12 repetitions, and 3–6 sets and 3–12 repetitions, respectively. For *(v) exercise tempo*, the ‘pause’ duration between eccentric and concentric phases differed in GPT3.5 (2/0/2), GPT4.0_INT_ (2/1/2), and GPT4.0_ADV_ (3/0/1/0, 2/0/1/0, and 1/0/1/0, in each phase). For *(vi), rest interval*, GPT3.5 proposed predominantly between 90–120-s, while GPT4.0_INT_ proposed 60–90-s (for high repetition exercise), 90–120-s (medium repetition), and 2–3-min (low-medium repetitions). In comparison, GPT4.0_ADV_ proposed 60–300-s rest intervals depending on training phase and exercise (main vs supplementary).

## DISCUSSION

ChatGPT generated relevant content for resistance training programming (Table 1). GPT3.5, GPT4.0_INT_ and GPT4.0_ADV_ proposed three 4-week phases (S1). Some subtle differences were observed between the responses in terms of exercise variables such as exercise selection, frequency, repetitions, and intensities. GPT4.0 (intermediate and advanced) provided additional information, reflecting a better understanding of training prescription. Furthermore, GPT4.0 was adept in tailoring training programmes to accommodate different resistance training competency levels. The generated training programmes generally considered training principles (e.g., progressive overload and variation), comparable with information contained within established guidelines and peer-reviewed resources, and articulated using standard academic language. These outputs indicated some degree of appropriate prioritisation by the technology, in terms of sourcing information.

Manipulation of volume and load intensity are key considerations in designing resistance training programmes [8] and exercise volume-induced changes in steroid hormones like testosterone and cortisol also influence strength gains [9, 10]. It is encouraging that both ChatGPT versions proposed three training phases with varying foci regarding training variables to facilitate strength development [11]. ChatGPT also incorporated both ‘main’ and ‘supplementary’ exercises, while proposing appropriate exercises that targeted major muscle groups, such as upper and lower body push-pull variations, which can stimulate hormonal responses that in turn facilitate muscular growth and strength [12]. ChatGPT also employed a split-routine to target specific muscle groups on separate days, a common practice in strength training [12]. The advanced programme proposed a block periodisation, which is common practice for well-trained individuals [12] and included unilateral (for engagement of stabilising muscles) and loaded bodyweight exercises. These routines and strategies adhere to the training principles (e.g., progressive overload, variation, specificity) that allows a training stimulus to remain optimal over time [12]. This prescription offers end-user more comprehensive and relevant information when prompted appropriately.

ChatGPT generally recommended 3–5 sessions of resistance training in a week (S1). GPT4.0_ADV_ stated that “four training sessions provides a balance between training volume and recovery for an advanced trainee” (S2). Higher training frequency (≥ 3 sessions/week) augments total weekly training volume, and positively impacts muscular strength [13]. The NSCA recommends 3–4 sessions/week for intermediate and 4–7 sessions/week for advanced trainers [12]. This frequency provides sufficient time for recovery and adaptation, whilst optimising hypertrophy and strength [14, 15]. Interestingly, only GPT4.0_ADV_ considered “active recovery” sessions, citing the promotion of blood flow and removal of waste products (S2). GPT models showed variable load intensity prescriptions, ranging from 60–95% (S1) to allow “proper progressive overload” (S2), with loads varied in main/supplementary exercises. These recommendations are aligned with conventional resistance training [12] but omit emerging trends like low-load prescriptions (< 60%) or blood flow restriction for muscle hypertrophy [8]. Varying repetition ranges for exercises were recommended by different versions of GPT, which generally, aligned with established research on muscle hypertrophy and strength development [12]. For example, the proposed heavy loads (> 85%) with fewer repetitions were specific to achieve training goals (e.g., maximal strength) (S1). Depending on the stated objective, GPT4.0_ADV_ proposed medium repetitions during the initial training period (accumulation), reducing to low-medium during “intensification”, and low during “realisation” phase. GPT3.5 proposed low-medium range repetitions (4–8 reps) for most exercises, while GPT4.0_INT_ proposed a relatively medium range (5–12 reps). Medium repetition ranges (e.g., 6–12 reps) may facilitate hypertrophy, and lower repetitions (e.g., 1–6 reps) enhance strength [12]. ChatGPT proposed a multiple-set system tailored to an individual’s training level for optimal strength gains (S1). Indeed, intermediate-level individuals benefit from a medium weekly dosage of 5–9 sets, while advanced individuals benefit from both medium and high (≥ 10 sets) weekly sets [14].

GPT4.0_ADV_ recommended additional volume and eccentric loading to enhance hypertrophy and prepare muscle tissues for heavier loads. Indeed, muscular strength can be optimised through training volume and “time under tension” or tempo [13, 16, 17]. As exercise tempo can affect training volume [14], differences in total time under tension, [e.g., 2/0/2 (GPT3.5) and 3/0/3/0 (e.g., GPT4.0_ADV_)] can possibly impact movement and influence training adaptation. Even though both eccentric and concentric training are necessary to optimise hypertrophy [8], current evidence supports strength training protocols with medium eccentric and fast concentric actions (e.g., 2-4/0/1/0) for optimising dynamic strength development in trained and untrained individuals [18]. ChatGPT did provide a debatable assertion that “muscle damage is a key driver for hypertrophy” (S2). These responses again reinforce the importance of human interpretation of responses, prior to application.

Prescribed rest intervals reflected the specific training goals (S1). For example, GPT4.0 (intermediate and advanced) contained appropriate rest interval durations of 60–300 s dependent on exercise repetitions, phase, and exercise types (S1). GPT3.5 proposed 90–120 s for all three phases, even when the training focus was strength development. This prescription deviates from the specificity concept to enhance training gains, which is likely suboptimal, given short rest intervals (e.g., 60–90 s) are usually applied to enhance hypertrophic responses [12] while longer rest intervals (2–5 min) facilitate greater recovery and enable heavier loads to be lifted [12]. Therefore, this indicates a more appropriate prescription from the latest GPT model, compared with earlier iterations.

Currently, ChatGPT supports autodidactic self-learning, but responses need to be carefully appraised. ChatGPT’s justifications such as “efficient” use of training time, as well as considerations of active recovery, nutrition, and hydration are noteworthy (S2), as these elements were not outlined in the prompts. This detail indicates a broader awareness of the subject matter than what was detailed in the prompt. A weekly routine that encompasses a well-rounded approach (including proper exercises, structured routines, adequate recovery etc.) is essential for optimising training effectiveness. Furthermore, ChatGPT delivered information in a language comparable to academic sources. However, the suggested guidelines (S1) and rationales (S2) appear to have overlooked some alternative training methods, loading strategies, and set configurations [19]. For example, the potential to induce substantial strength gains through cluster sets, variable resistance training, and blood flow restriction. Other methods, such as supersets and drop sets, which are time-efficient and effective to induce strength gains, were also omitted. This exclusion indicates a possible lack of alignment with contemporary, evolving training methodologies. Future prompts may need to be refined to consider emerging research and suggested programmes should be scrutinised by a topic expert. While only male responses are reported, the prompts for a female subject received comparable recommendations to the male subject regarding training phases, weekly structure, and session routines. This lack of distinction may be due to the disproportionately low number of female training studies and source material available for ChatGPT to draw upon. We strongly support future research focused on female training programmes and acknowledge that females may require different prescription needs to males [20].

In this article, we exclusively examined artificial intelligence-generated training programmes, while thoroughly considering literature and guidelines, leaving the potential impact of personal trainer’s recommendations and supervisions unassessed. Also, we did not explore whether ChatGPT can synthesise contextual information, for example, modifying training based on physical readiness. Nevertheless, ChatGPT offered no potential real-time adjustments, or revisions to training protocols, based on feedback or individual progression. Intuitively, practitioners with a sound understanding of resistance training remain best suited to adjusting these variables. Currently, we propose that ChatGPT cannot replace the judgement and empathy of a human practitioner and gaps in acknowledging recent advancements in related research are evident.

## CONCLUSIONS

ChatGPT generated realistic information for resistance training, guided by user prompts. However, the suggested programme may require modification. GPT4.0_ADV_ provided greater detail and consideration of training status when prescribing training. As artificial intelligence technologies develop over time, future versions may enhance the user experience. Further exploration and validation of ChatGPT-generated training programmes in real-world settings and with actual athletes is warranted to ascertain their practical utility.

### Practical Applications

– ChatGPT can accelerate idea generation and detailed resistance training prescription.
– ChatGPT produced credible information, which may be suitable for general exercise guidance. However, additional professional assistance appears necessary for optimal outcomes.
– During isolating circumstances such as the COVID-19 pandemic, the use of a ChatGPT ‘chatbot’ for training prescription may help bridge information gaps.
– ChatGPT should be used as a supplementary tool (not a replacement) and combining artificial intelligence with human expertise may optimise exercise prescription effectiveness.

## Supplementary Material

Artificial intelligence in sport: Exploring the potential of using ChatGPT in resistance training prescription
