# Supplementary material for: Artificial intelligence in sport: Exploring the potential of using ChatGPT in resistance training prescription
Source: Biol Sport. 2023 Nov 20;41(2):209–20. doi: 10.5114/biolsport.2024.132987 (PMC10955742; doi:10.5114/biolsport.2024.132987)
Supplement: Artificial intelligence in sport: Exploring the potential of using ChatGPT in resistance training prescription [file JBS-41-51817-s1.pdf]

SUPPLEMENTARY FILE 1. ChatGPT generated texts for the 12 weeks intermediate and advanced resistance training programmes.

GPT3.5	GPT4.0	GPT4.0
**Intermediate programme**	**Intermediate programme**	**Advanced programme**
**PROMPT 1**	**PROMPT 1**	**PROMPT 1**
**Weeks 1–4: Foundation Phase**	**Weeks 1–4 (High Volume)**	**Weeks 1–4 (Accumulation Phase)**
		(Increasing volume)
Day 1: Upper Body	Day 1 (Chest, Shoulders, Triceps):	Frequency: 4 days a week.
Bench Press: 3 sets of 6–8 reps	Bench Press: 3 sets of 12 reps	
Bent-over Rows: 3 sets of 6–8 reps	Overhead Press: 3 sets of 12 reps	Day 1: Lower Body
Overhead Shoulder Press: 3 sets of 6–8 reps	Dumbbell Fly: 3 sets of 12 reps	Squat – 4 sets × 8 reps
Bicep Curls: 2 sets of 10 reps	Lateral Raise: 3 sets of 12 reps	Deadlift – 3 sets × 8 reps
Tricep Dips: 2 sets of 10 reps	Tricep Pushdown: 3 sets of 12 reps	Bulgarian Split Squats – 3 sets × 10 reps per leg
		Romanian Deadlifts – 3 sets × 10 reps
Day 2: Lower Body	Day 2 (Legs, Abs):	Calf Raises – 4 sets × 12 reps
Squats: 3 sets of 6–8 reps	Squat: 3 sets of 12 reps	
Deadlifts: 3 sets of 6–8 reps	Deadlift: 3 sets of 12 reps	Day 2: Upper Body
Leg Press: 3 sets of 8–10 reps	Leg Press: 3 sets of 12 reps	Bench Press – 4 sets × 8 reps
Leg Curls: 2 sets of 10 reps	Calf Raises: 3 sets of 12 reps	Barbell Rows – 4 sets × 8 reps
Calf Raises: 2 sets of 12 reps	Plank: 3 sets of 60 seconds	Overhead Press – 3 sets × 10 reps
		Pull-Ups – 3 sets × 8 reps (add weight if necessary)
**PROMPT 2**	Day 3 (Rest)	Dips – 3 sets × 10 reps (add weight if necessary)
Day 1: Upper Body	Day 4 (Back, Biceps):	Day 3: Rest or Active Recovery
Load: 70–75%; Rest: 2 mins; Tempo: 2:0:2	Pull-up: 3 sets of 12 reps	(e.g., light cardio, stretching)
Load: 70–75%; Rest: 2 mins; Tempo: 2:0:2	Bent Over Row: 3 sets of 12 reps	
Load: 70–75%; Rest: 2 mins; Tempo: 2:0:2	Lat Pulldown: 3 sets of 12 reps	Day 4: Lower Body
Load: 60–65%; Rest: 1.5 mins; Tempo: 2:0:2	Barbell Curl: 3 sets of 12 reps	Front Squat – 3 sets × 8 reps
Load: Bodyweight; Rest: 1.5 mins; Tempo: 2:0:2	Hammer Curl: 3 sets of 12 reps	Sumo Deadlift – 3 sets × 8 reps
		Lunges – 3 sets × 10 reps per leg
Day 2: Lower Body	Day 5 (Rest)	Glute Bridge – 3 sets × 12 reps
Load: 70–75%; Rest: 2 mins; Tempo: 2:0:2		Seated Calf Raise – 4 sets × 12 reps
Load: 70–75%; Rest: 2 mins; Tempo: 2:0:2	Day 6 & 7 (Repeat Day 1 & 2)	
Load: 65–70%; Rest: 1.5 mins; Tempo: 2:0:2		Day 5: Upper Body
Load: 60–65%; Rest: 1.5 mins; Tempo: 2:0:2	**PROMPT 2**	Incline Bench Press – 4 sets × 8 reps
Load: 60–65%; Rest: 1 min; Tempo: 2:0:2		Pull-Ups (wide grip) – 4 sets × 8 reps
	Load intensity: 60–70% of one-rep max (1RM)	Dumbbell Shoulder Press – 3 sets × 10 reps
	Rest interval: 60–90 seconds between sets	Barbell Curl – 3 sets × 10 reps
	Exercise tempo: 2–1–2 (2 seconds for the eccentric or lowering phase, 1 second pause, 2 seconds for the concentric or lifting phase)	Skull Crushers – 3 sets × 10 reps<br><br>Day 6 & 7: Rest or Active Recovery
	Day 1 (Chest, Shoulders, Triceps):	**PROMPT 2**
	Bench Press: 3 sets of 12 reps	
	Overhead Press: 3 sets of 12 reps	Intensity: ~70–80% of 1RM
	Dumbbell Fly: 3 sets of 12 reps	
	Lateral Raise: 3 sets of 12 reps	Rest Interval: 60–90 seconds between sets
	Tricep Pushdown: 3 sets of 12 reps	
		Tempo: 3–0–1–0 (3 seconds eccentric, 0 pause at the bottom, 1 second concentric, 0 pause at the top)
	Repeat for days 2–7 as above.	

SUPPLEMENTARY FILE 1. Continue.

GPT3.5	GPT4.0	GPT4.0
**Intermediate programme**	**Intermediate programme**	**Advanced programme**
***Cont.* PROMPT 1**<br><br>**Weeks 5–8: Strength Building Phase**<br><br>Day 1: Upper Body<br>Bench Press: 4 sets of 5–6 reps<br>Pull-Ups/Assisted Pull-Ups: 4 sets of 6–8 reps<br>Seated Shoulder Press: 4 sets of 5–6 reps<br>Barbell Bicep Curls: 3 sets of 8 reps<br>Skull Crushers: 3 sets of 8 reps<br><br>Day 2: Lower Body<br>Back Squats: 4 sets of 5–6 reps<br>Romanian Deadlifts: 4 sets of 5–6 reps<br>Leg Press: 3 sets of 6–8 reps<br>Leg Extensions: 3 sets of 8 reps<br>Standing Calf Raises: 3 sets of 10 reps	***Cont.* PROMPT 1**<br><br>**Weeks 5–8 (Moderate Volume/High Intensity)**<br><br>Increase weights and lower reps to focus on strength.<br><br>Day 1 (Chest, Shoulders, Triceps):<br>Bench Press: 4 sets of 8 reps<br>Overhead Press: 4 sets of 8 reps<br>Dumbbell Fly: 4 sets of 8 reps<br>Lateral Raise: 4 sets of 8 reps<br>Tricep Pushdown: 4 sets of 8 reps<br><br>Day 2 (Legs, Abs):<br>Squat: 4 sets of 8 reps<br>Deadlift: 4 sets of 8 reps<br>Leg Press: 4 sets of 8 reps<br>Calf Raises: 4 sets of 8 reps<br>Plank: 4 sets of 60 seconds<br><br>Day 3 (Rest)<br><br>Day 4 (Back, Biceps):<br>Pull-up: 4 sets of 8 reps<br>Bent Over Row: 4 sets of 8 reps<br>Lat Pulldown: 4 sets of 8 reps<br>Barbell Curl: 4 sets of 8 reps<br>Hammer Curl: 4 sets of 8 reps<br><br>Day 5 (Rest)<br><br>Day 6 & 7 (Repeat Day 1 & 2)	***Cont.* PROMPT 1**<br><br>**Weeks 5–8: Intensification Phase**<br>(Increasing intensity)<br><br>Frequency: 4 days a week.<br><br>Day 1: Lower Body<br>Squat – 5 sets × 5 reps<br>Deadlift – 4 sets × 5 reps<br>Bulgarian Split Squats – 4 sets × 8 reps per leg<br>Romanian Deadlifts – 4 sets × 8 reps<br>Calf Raises – 5 sets × 10 reps<br><br>Day 2: Upper Body<br>Bench Press – 5 sets × 5 reps<br>Barbell Rows – 5 sets × 5 reps<br>Overhead Press – 4 sets × 6 reps<br>Weighted Pull-Ups – 4 sets × 6 reps<br>Weighted Dips – 4 sets × 8 reps<br><br>Day 3: Rest or Active Recovery<br><br>Day 4: Lower Body<br>Front Squat – 4 sets × 6 reps<br>Sumo Deadlift – 4 sets × 6 reps<br>Lunges – 4 sets × 8 reps per leg<br>Glute Bridge – 4 sets × 10 reps<br>Seated Calf Raise – 5 sets × 10 reps<br><br>Day 5: Upper Body<br>Incline Bench Press – 5 sets × 5 reps<br>Pull-Ups (wide grip) – 5 sets × 6 reps<br>Dumbbell Shoulder Press – 4 sets × 6 reps<br>Barbell Curl – 4 sets × 8 reps<br>Skull Crushers – 4 sets × 8 reps<br><br>Day 6 & 7: Rest or Active Recovery
***Cont.* PROMPT 2**<br>Day 1: Upper Body<br>Load: 75–80%; Rest: 2 mins; Tempo: 2:0:2<br>Load: Bodyweight; Rest: 2 mins; Tempo: 2:0:2<br>Load: 75–80%; Rest: 2 mins; Tempo: 2:0:2<br>Load: 65–70%; Rest: 1.5 mins; Tempo: 2:0:2<br>Load: 65–70%; Rest: 1.5 mins; Tempo: 2:0:2<br><br>Day 2: Lower Body<br>Load: 75–80%; Rest: 2 mins; Tempo: 2:0:2<br>Load: 75–80%; Rest: 2 mins; Tempo: 2:0:2<br>Load: 70–75%; Rest: 1.5 mins; Tempo: 2:0:2<br>Load: 65–70%; Rest: 1.5 mins; Tempo: 2:0:2<br>Load: 65–70%; Rest: 1 min; Tempo: 2:0:2	***Cont.* PROMPT 2**<br><br>Load intensity: 70–80% of 1RM<br>Rest interval: 90–120 seconds between sets<br>Exercise tempo: 2–0–2<br><br>Day 1 (Chest, Shoulders, Triceps):<br>Bench Press: 4 sets of 8 reps<br>Overhead Press: 4 sets of 8 reps<br>Dumbbell Fly: 4 sets of 8 reps<br>Lateral Raise: 4 sets of 8 reps<br>Tricep Pushdown: 4 sets of 8 reps<br><br>Repeat for days 2–7 as above.	***Cont.* PROMPT 2**<br><br>Intensity: ~80–90% of 1RM<br><br>Rest Interval: 2–3 minutes between sets<br><br>Tempo: 2–0–1–0

SUPPLEMENTARY FILE 1. Continue.

GPT3.5	GPT4.0	GPT4.0
**Intermediate programme**	**Intermediate programme**	**Advanced programme**
***Cont.* PROMPT 1**	***Cont.* PROMPT 1**	***Cont.* PROMPT 1**
**Weeks 9–12: Intensification Phase**	**Weeks 9–12 (Low Volume/Very High Intensity)**	**Weeks 9–12: Realization Phase**
		(Testing new strength levels)
Day 1: Upper Body	Further increase weights and decrease reps to peak strength.	
Incline Bench Press: 4 sets of 4–5 reps		Frequency: 4 days a week.
Bent-over Rows (Barbell/D'bell): 4 sets of 4–5 reps		
Arnold Press: 3 sets of 6 reps	Day 1 (Chest, Shoulders, Triceps):	Day 1: Lower Body
Barbell Curls: 3 sets of 6 reps	Bench Press: 5 sets of 5 reps	Squat – 6 sets × 3 reps
Tricep Rope Pushdowns: 3 sets of 6 reps	Overhead Press: 5 sets of 5 reps	Deadlift – 5 sets × 3 reps
	Dumbbell Fly: 5 sets of 5 reps	Bulgarian Split Squats – 4 sets × 6 reps per leg
Day 2: Lower Body	Lateral Raise: 5 sets of 5 reps	Romanian Deadlifts – 4 sets × 6 reps
Front Squats: 4 sets of 4–5 reps	Tricep Pushdown: 5 sets of 5 reps	Calf Raises – 5 sets × 8 reps
Sumo Deadlifts: 4 sets of 4–5 reps		
Bulgarian Split Squats: 3 sets of 6 reps per leg	Day 2 (Legs, Abs):	Day 2: Upper Body
Hamstring Curls: 3 sets of 6 reps	Squat: 5 sets of 5 reps	Bench Press – 6 sets × 3 reps
Seated Calf Raises: 3 sets of 8 reps	Deadlift: 5 sets of 5 reps	Barbell Rows – 6 sets × 3 reps
	Leg Press: 5 sets of 5 reps	Overhead Press – 5 sets × 4 reps
***Cont.* PROMPT 2**	Calf Raises: 5 sets of 5 reps	Weighted Pull-Ups – 5 sets × 4 reps
	Plank: 5 sets of 60 seconds	Weighted Dips – 5 sets × 6 reps
Day 1: Upper Body		
Load: 80–85%; Rest: 2 mins; Tempo: 2:0:2	Day 3 (Rest)	Day 3: Rest or Active Recovery
Load: 80–85%; Rest: 2 minutes; Tempo: 2:0:2		
Load: 70–75%; Rest: 1.5 mins; Tempo: 2:0:2	Day 4 (Back, Biceps):	Day 4: Lower Body
Load: 70–75%; Rest: 1.5 mins; Tempo: 2:0:2	Pull-up: 5 sets of 5 reps	Front Squat – 5 sets × 4 reps
Load: 70–75%; Rest: 1.5 mins; Tempo: 2:0:2	Bent Over Row: 5 sets of 5 reps	Sumo Deadlift – 5 sets × 4 reps
	Lat Pulldown: 5 sets of 5 reps	Lunges – 4 sets × 6 reps per leg
Day 2: Lower Body	Barbell Curl: 5 sets of 5 reps	Glute Bridge – 4 sets × 8 reps
Load: 80–85%; Rest: 2 mins; Tempo: 2:0:2	Hammer Curl: 5 sets of 5 reps	Seated Calf Raise – 5 sets × 8 reps
Load: 80–85%; Rest: 2 mins; Tempo: 2:0:2		
Load: 70–75%; Rest: 1.5 mins; Tempo: 2:0:2	Day 5 (Rest)	Day 5: Upper Body
Load: 70–75%; Rest: 1.5 mins; Tempo: 2:0:2		Incline Bench Press – 6 sets × 3 reps
Load: 65–70%; Rest: 1 min; Tempo: 2:0:2	Day 6 & 7 (Repeat Day 1 & 2)	Pull-Ups (wide grip) – 6 sets × 4 reps
		Dumbbell Shoulder Press – 5 sets × 4 reps
		Barbell Curl – 5 sets × 6 reps
	***Cont.* PROMPT 2**	Skull Crushers – 5 sets × 6 reps
	Load intensity: 80–90% of 1RM	Day 6 & 7: Rest or Active Recovery
	Rest interval: 2–3 minutes between sets	
	Exercise tempo: 1–0–1	***Cont.* PROMPT 2**
	Day 1 (Chest, Shoulders, Triceps):	Intensity: ~90–95% of 1RM for main lifts, ~80–85% for accessory lifts
	Bench Press: 5 sets of 5 reps	
	Overhead Press: 5 sets of 5 reps	Rest Interval: 3–5 minutes for main lifts, 2–3 minutes for accessory lifts
	Dumbbell Fly: 5 sets of 5 reps	
	Lateral Raise: 5 sets of 5 reps	
	Tricep Pushdown: 5 sets of 5 reps	Tempo: 1–0–1–0
	Repeat for days 2–7 as above.	
**Remarks**	**Remarks**	**Remarks**

SUPPLEMENTARY FILE 1. Continue.

GPT3.5	GPT4.0	GPT4.0
**Intermediate programme**	**Intermediate programme**	**Advanced programme**
***Cont.* PROMPT 1 & 2**	***Cont.* PROMPT 1**	***Cont.* PROMPT 1**
General Guidelines:<br>– Perform the program 3 days a week, with at least one rest day between sessions.<br>– Increase the weight when you can complete the recommended reps and sets with proper form.<br>– Rest 2–3 minutes between sets of compound exercises and 1–2 minutes for isolation exercises.<br>– Focus on proper form and technique to prevent injuries and maximize gains.<br>– Incorporate a 5–10 minute warm-up and cool-down routine before and after each workout.<br>Track your progress to monitor improvements and make necessary adjustments.<br><br>Remember, adequate nutrition and sufficient rest are essential for recovery and muscle growth. Listen to your body, and if you ever experience pain or discomfort, consult with a fitness professional or healthcare provider.	Note:<br>Always start each workout with a good warm-up, and finish the workout with a cool-down. Adjust weights as needed to ensure they're challenging but don't compromise form. Ensure rest days are taken to promote muscle recovery and growth.<br><br>Lastly, it's essential to pair this program with a balanced diet that supports your training goals. Please consult with a fitness professional to ensure that these exercises and the program are suitable for your particular circumstances.<br><br>Before starting any new exercise program, it's always a good idea to consult with a healthcare professional.<br><br>***Cont.* PROMPT 2**<br><br>Note: The 1RM should be updated periodically, possibly every 4 weeks, to account for strength increases. Exercise tempo should be adjusted according to individual comfort and safety. Always ensure proper form to prevent injury. Remember that recovery is crucial – do not skip rest days. Consult a professional for individual advice.<br><br>Again, ensure that you're using proper form and technique throughout the program, and if you have any concerns or questions, consult with a certified fitness professional or healthcare provider.	Note:<br>Warm-up appropriately before each session.<br>Increase the weight if the prescribed reps feel easy.<br>Ensure proper form and technique.<br>Consider adding in supplemental work (abdominal exercises, rotator cuff exercises) and stretching/mobility work on active recovery days.<br>Ensure adequate nutrition and sleep to support recovery.<br>After this 12–week program, you may want to consider taking a deload week, where volume and intensity are reduced to allow for optimal recovery before starting another program or cycle.<br><br>***Cont.* PROMPT 2**<br><br>These percentages are starting points. It's essential to listen to one's body and adjust based on how the training feels. If someone finds that a set at a particular percentage feels too easy or hard, adjusting the weight accordingly is important. Proper warm-up, form, and technique are paramount, especially when working with higher intensity loads.

Note: All the texts within this table were generated by ChatGPT. During prompt 2, GPT-3.5 provided the same training programme as prompt 1, which we removed to improve readability, and only the additional variables were shown. GPT-3.5 also provided the same "general guidelines" in prompt 1 and prompt 2. *Training tempo:* a sequence 3:1:2:0 (or 3/1/2/0 or 3-1-2-0) reflects an exercise performed as 3 seconds eccentric, 1 second pause before the next motion, 2 seconds concentric, and 0 or no pause before the next repetition. *Abbreviations:* GPT: Generative Pre-trained Transformer; rep/reps: repetition(s); mins: minutes.

SUPPLEMENTARY FILE 2. GPT3.5 and GPT4.0 generated text specifying reasons of the suggested programme variables.

Intermediate training programme	Intermediate training programme	Advanced training programme
**GPT3.5**	**GPT4.0**	**GPT4.0**
Foundation Phase (Weeks 1–4)<br>– 6–8 reps: Moderate rep range to build a foundation of strength and muscular endurance.<br>– 70–75% Load Intensity: A balance between challenging the muscles and allowing for proper progression.<br>– 2 minutes Rest Interval: Sufficient time to recover between sets and perform the exercises with adequate intensity.<br>– Tempo: 2:0:2 (2 seconds eccentric, 0–second pause, 2 seconds concentric): Emphasizes controlled movements and muscle tension.<br><br>Strength Building Phase (Weeks 5–8)<br>– 5–6 reps: Lower rep range for increased intensity to promote strength gains.<br>– 75–80% Load Intensity: Higher intensity to challenge the muscles further as strength improves.<br>– 2 minutes Rest Interval: Sufficient rest for heavy lifting and recovery between sets.<br>– Tempo: 2:0:2: Slower eccentric phase enhances time under tension for greater strength development.<br><br>Intensification Phase (Weeks 9–12)<br>– 4–5 reps: Lower rep range to focus on maximal strength development.<br>– 80–85% Load Intensity: High intensity to stimulate strength gains in the intermediate lifter.<br>– 2 minutes Rest Interval: Allows for proper recovery between heavy sets.<br>Tempo: 2:0:2: Controlled tempo to maintain muscle tension during the challenging lifts.	1. Periodisation<br>Periodisation is used to structure training into different phases over the course of the 12 weeks. Each phase focuses on different aspects of strength training (high volume, moderate intensity, and high intensity), allowing for gradual adaptation and reducing the risk of overtraining.<br><br>2. High Volume (Weeks 1–4)<br><br>Lower intensity (60–70% 1RM) and higher volume (more repetitions) is used initially to build muscular endurance, improve technique, and prepare the body for the higher intensities to come.<br><br>3. Moderate Volume/High Intensity (Weeks 5–8)<br><br>The intensity is increased to 70–80% of 1RM and volume is reduced to allow for greater strength and muscle size gains, and to further prepare the body for the very high intensity in the final phase.<br><br>4. Low Volume/Very High Intensity (Weeks 9–12)<br><br>In this phase, intensity is further increased to 80–90% of 1RM and volume is further reduced. This focuses on maximizing muscle strength.<br><br>5. Rest Intervals:<br><br>Shorter rest intervals are used during the high-volume phase to keep the intensity moderate and ensure the body is adequately stressed by the higher number of reps.<br>As the program transitions to higher intensity, the rest periods between sets are extended to allow for sufficient recovery and enable the individual to lift more weight.	1. Periodization:<br>Reason: Allows for systematic programming to avoid plateaus and manage fatigue.<br>Benefits: Maximizes adaptation, minimizes overtraining, and allows for targeted training outcomes (e.g., hypertrophy, strength, power).<br><br>2. Load Intensity (% of 1RM):<br>Weeks 1–4 (70–80%):<br>Reason: Focus on moderate weight to allow for higher volume and focus on technique.<br>Benefits: Lays a foundation, promoting hypertrophy and preparing tissues for heavier loads.<br><br>Weeks 5–8 (80–90%):<br>Reason: Increase in weight to shift focus towards pure strength adaptations.<br>Benefits: Neuromuscular adaptations, increased motor unit recruitment.<br><br>Weeks 9–12 (90–95%):<br>Reason: Heavy weights to realize strength gains and test new strength levels.<br>Benefits: Peak strength adaptation and increased CNS efficiency.<br><br>3. Rest Intervals:<br>Weeks 1–4 (60–90 seconds):<br>Reason: Shorter rest due to moderate loads; sufficient for ATP-PCr energy system recovery.<br>Benefits: Enhances hypertrophic adaptations and keeps workout duration reasonable.<br><br>Weeks 5–8 & 9–12 (2–5 minutes):<br>Reason: As intensity increases, more recovery time is needed between sets.<br>Benefits: Allows for full recovery and maximal effort in subsequent sets, optimizing strength gains.

SUPPLEMENTARY FILE 2. Continue.

Intermediate training programme	Intermediate training programme	Advanced training programme
**GPT3.5**	**GPT4.0**	**GPT4.0**
	6. Exercise Tempo:<br><br>A slower tempo (2–1–2) is used during the high volume phase to increase time under tension, improving muscular endurance and promoting technique.<br>As the program progresses to higher intensities, the tempo is increased slightly to allow for heavier weights to be lifted.<br><br>7. Exercise Selection:<br><br>The program includes compound movements, which work multiple muscle groups at once. This is efficient and effective for improving overall strength.<br>Exercise order is arranged in a way that larger muscle groups are worked before smaller ones.<br><br>8. 1RM Updates:<br><br>Regularly updating the 1RM ensures the training load is adjusted as strength increases. This keeps the intensity appropriate for the desired training effect.<br><br>9. Rest and Recovery:<br><br>Rest days are crucial for muscle recovery and growth. They also prevent overtraining and reduce the risk of injuries.<br><br>Remember, while this plan provides a general guideline, it should be individualized to meet specific needs and goals. The person should also consider their diet, hydration, and sleep as these factors greatly affect the outcome of the training program. Always consult a professional to ensure a safe and effective training plan.	4. Tempo (eccentric-pause-concentric-pause):<br>Weeks 1–4 (3–0–1–0):<br>Reason: Emphasis on eccentric phase promotes muscle damage, a key driver for hypertrophy.<br>Benefits: Maximizes time under tension (TUT) and promotes hypertrophy.<br><br>Weeks 5–8 & 9–12 (2–0–1–0 & 1–0–1–0):<br>Reason: As weight increases, it's challenging to maintain a slow eccentric; focus shifts to moving the weight effectively.<br>Benefits: Allows for handling of heavier loads and optimal force production.<br><br>5. Exercise Selection:<br>Reason: Compound movements are prioritized as they recruit multiple muscle groups, allowing for heavier loads and maximizing strength adaptations.<br>Benefits: Functional strength gains, comprehensive muscle development, and efficient use of training time.<br><br>6. Frequency (4 days a week):<br>Reason: Provides a balance between training volume and recovery for an advanced trainee.<br>Benefits: Allows sufficient stimuli for adaptation while ensuring adequate recovery, reducing the risk of overtraining.<br><br>7. Active Recovery Days:<br>Reason: Active recovery can promote blood flow and aid in the removal of waste products.<br>Benefits: Speeds up recovery, mitigates soreness, and reduces the risk of overuse injuries.<br>These variables, when combined systematically, aim to provide an effective platform for advanced strength adaptations while managing fatigue and injury risk.

Note: All the texts within this table were generated by ChatGPT. *Training tempo:* a sequence 3:1:2:0 (or 3/1/2/0 or 3-1-2-0) reflects an exercise performed as 3 seconds eccentric, 1 second pause before the next motion, 2 seconds concentric, and 0 or no pause before the next repetition. *Abbreviations:* GPT: Generative Pre-trained Transformer; rep/reps: repetition(s); mins: minutes;
